# The impact of intraoperative blood pressure variability on the risk of postoperative adverse outcomes in non-cardiac surgery: a systematic review

**DOI:** 10.1007/s00540-022-03035-w

**Published:** 2022-01-13

**Authors:** Zbigniew Putowski, Marcelina Czok, Łukasz J. Krzych

**Affiliations:** 1 Students’ Scientific Society, Department of Anaesthesiology and Intensive Care, Faculty of Medical Sciences in Katowice, Medical University of Silesia, Medyków 14 Street, 40752 Katowice, Poland; 2 Department of Anaesthesiology and Intensive Care, Faculty of Medical Sciences in Katowice, Medical University of Silesia, Katowice, Poland

**Keywords:** Variability, Blood pressure, Intraoperative period, Postoperative complications

## Abstract

Hemodynamic stability during surgery seems to account for positive postoperative outcomes in patients. However, little is known about the impact of intraoperative blood pressure variability (IBPV) on the postoperative complications. The aim was to investigate whether IBPV is associated with the development of postoperative complications and what is the nature of this association. We conducted a systematic search in PubMed, Medical Subject Headings, Embase, Web of Science, SCOPUS, clinicaltrials.gov, and Cochrane Library on the 8th of April, 2021. We included studies that only focused on adults who underwent primarily elective, non-cardiac surgery in which intraoperative blood pressure variation was measured and analyzed in regard to postoperative, non-surgical complications. We identified 11 papers. The studies varied in terms of applied definitions of blood pressure variation, of which standard deviation and average real variability were the most commonly applied definitions. Among the studies, the most consistent analyzed outcome was a 30-day mortality. The studies presented highly heterogeneous results, even after taking into account only the studies of best quality. Both higher and lower IBPV were reported to be associated for postoperative complications. Based on a limited number of studies, IBPV does not seem to be a reliable indicator in predicting postoperative complications. Existing premises suggest that either higher or lower IBPV could contribute to postoperative complications. Taking into account the heterogeneity and quality of the studies, the conclusions may not be definitive.

**Supplementary Information:**

The online version contains supplementary material available at 10.1007/s00540-022-03035-w.

## Introduction

Intraoperative hemodynamic homeostasis remains one of the crucial factors accounting for the postoperative prognosis of patients undergoing either cardiac or non-cardiac surgery [1]. Blood pressure monitoring stands as a cornerstone method for assessing perfusion during surgery [2]. The relationship between intraoperative hypotension (IOH) and postoperative complications received much attention during the recent years; however, there are still various aspects of intraoperative blood pressure that could enhance our understanding of tissue perfusion [3]. One of these aspects is the intraoperative blood pressure variability (IBPV). IBPV is a continuous variable that describes the degree of variation of a set of blood pressure measurements. It can be expressed in various forms, an example of which is the standard deviation (SD). On one hand, too high IBPV could reflect hemodynamic instability, i.e., successive changes in blood pressure would result in simultaneous perfusion disturbances (even without directly experiencing hypo- or hypertension). On the other hand, too low IBPV could reflect a patient's inability to adapt to changing hemodynamic circumstances (e.g., persistent hypotension despite adequate fluid infusion or vasopressor support would result in low IBPV). Moreover, in 2019, the Perioperative Quality Initiative (POQI) consensus on intraoperative blood pressure cited only one paper that focused on IBPV [1]. Due to seemingly limited data on the above-mentioned issue and unclear physiological implications, we decided to collect and analyze all the available data regarding this interesting issue in a systematic manner. Thus, this review aimed to answer the following question: If IBPV impacts postoperative outcomes, what is the nature of this association?

## Methods

This study was conducted in accordance with the PRISMA 2020 checklist [4].

### Eligibility criteria

We included studies that only focused on adults who underwent primarily elective, non-cardiac surgery in which IBPV was measured and analyzed in regard to postoperative, non-surgical complications. We included papers of which full reports were published before the day of search. Additionally, the papers had to be published in English, regardless of the year of publication. Studies were excluded when they selected a subgroup of patients with a specific comorbidity that was not part of the reason to perform the surgical procedure. Additionally, cardiac surgery papers, papers that included only emergency procedures, case reports, case series, systematic reviews, and meta-analyses were excluded. Studies in which blood pressure measurements were performed in time intervals longer than 5 min were not taken into account as well, as in such cases IBPV could have been omitted in a more significant matter.

### Information sources

The search was conducted within PubMed, Medical Subject Headings, Web of Science, SCOPUS, clinicaltrials.gov, Embase, and Cochrane Library on the 8th of April, 2021.

### Search

Search string for PubMed: (((intraoperative OR intraoperatively OR perioperative OR perioperatively) AND (variation OR lability OR variability OR deviation OR coefficient of variation OR fluctuation)) AND (myocardial injury OR major adverse cerebrovascular cardiovascular events OR postoperative complication* OR adverse outcome* OR cardiac OR renal OR organ injury OR organ dysfunction OR acute kidney injury OR myocardial infarction OR stroke OR death OR mortality OR length of stay OR cerebral OR complication OR adverse event* OR ischaem* OR injury OR delirium OR cerebrovascular OR coronary OR LOS OR accident)) AND (blood pressure OR systolic blood pressure OR diastolic blood pressure OR mean arterial pressure).

The remaining search strings are available in the Supplementary material 1.

### Study selection and data collection process

After importing all the papers from the initial search using search string, two independent investigators assessed studies by analyzing titles and abstracts (via Mendeley^®^). This study was processed further if all adjudicators (ZP and MC) agreed to include the paper for review. If only one reviewer agreed to proceed with the manuscript, the second assessment of the paper was performed by the third investigator (ŁJK).

### Data items

Authors, year of publication, type of a study, patients’ characteristics, type of surgery, intraoperative blood pressure variability, and postoperative complications were outcomes.

### Quality assessment

Newcastle–Ottawa scale (NOS) was implemented to assess the quality of the included studies [5]. The total NOS score of each study was converted to Agency for Healthcare Research and Quality standards [6]. In regard to the “comparability” criteria, we considered that the most important variable that needed to be controlled for was the occurrence of IOH. Two independent investigators (ZP and MC) performed the quality assessment and then, any differences were resolved by a discussion and the final decision was accepted by ŁJK.

## Results

### Included studies

**Fig. 1:**
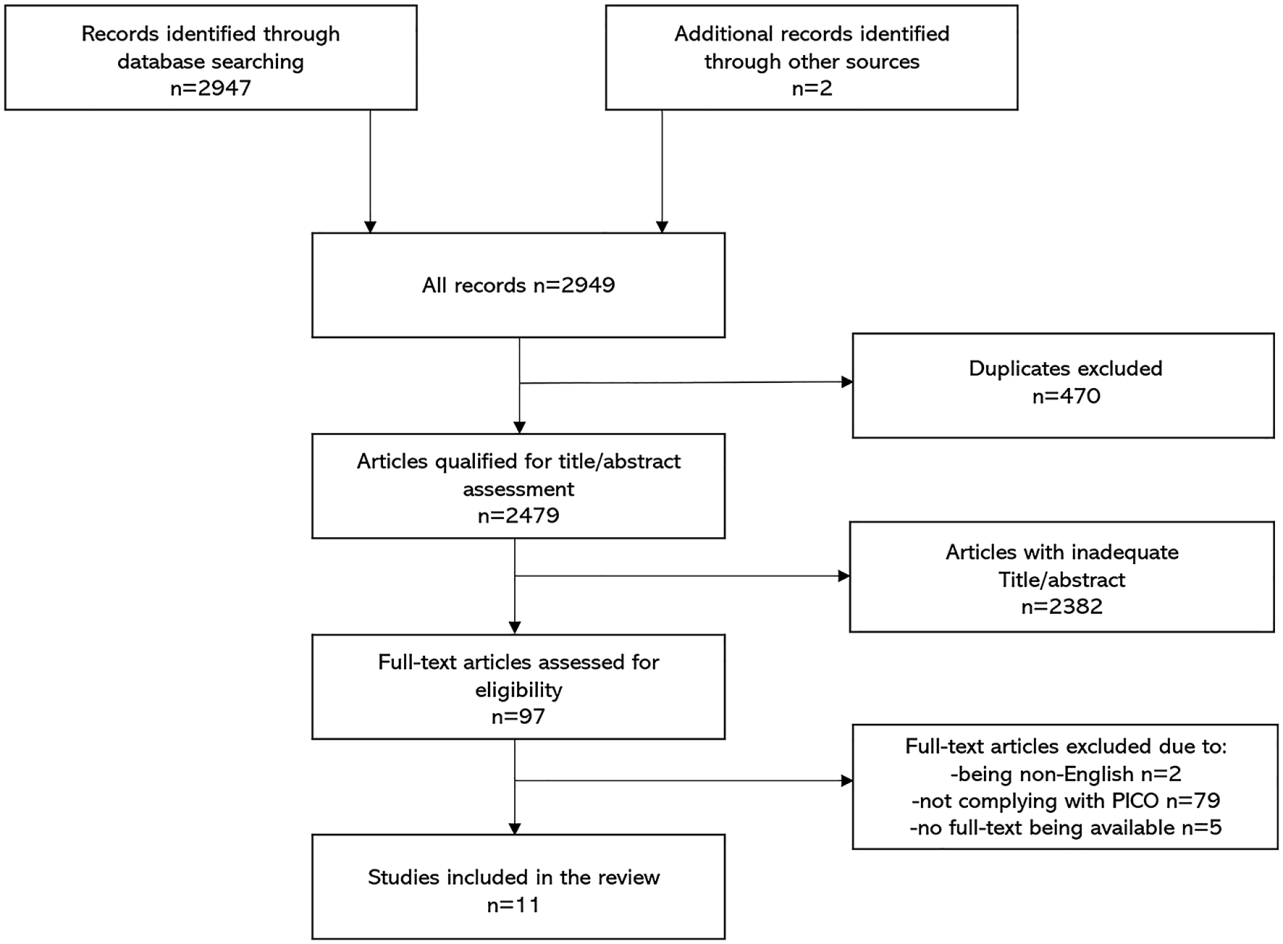
Flowchart of the progress of retrieved reports through the review

By using the search string within various medical databases (look at *Information sources*), we identified 2949 articles in total. After removing duplicates (*n* = 470), we screened the remaining papers by evaluating titles and abstracts (*n* = 2479). By using the PICO criteria and the inclusion and the exclusion criteria, we distinguished 97 papers for the full-text read assessment. After excluding the articles for numerous reasons (non-English papers = 2, papers not complying with the PICO criteria = 79, and papers with no full-text available = 5), the final 11 papers were included in the systematic review. There were 9 cohort studies (6 retrospective [7, 8, 12, 14, 15, 17] 3 prospective studies [11, 13, 16]) and 2 case–control studies [9, 10]. Study selection process is presented on a flowchart (Fig. 1).

### Quality assessment

By implementing NOS, we assessed all 11 studies (of which 9 were cohort studies and 2 were case–control studies). Overall, we identified 4 studies of “good” quality [11, 12, 15, 17] and 7 of “poor” quality (Supplementary material 2) [7–10, 13, 14, 16]. The quality of the latter studies suffered from the lack of adequate controlling for confounding factors, as these studies did not control for IOH. In general, there were no systematic problems regarding “selection” and “outcomes” criteria.

### Patient characteristics

The number of patients varied from 33 to 104,401, with a median of 917 patients (Table 1 and Supplementary material 3) [7–17]. The mean or the median age of participants in 7 studies was below 65 years [10, 12–17], whereas in the remaining 4, above 65 years [7–9, 11]. Information regarding gender was available in 9 studies [7–10, 12–17], of which 6 had a similar gender ratio of about 40–60% [7, 8, 10, 12, 15, 17]. In 4 studies, all patients underwent general anesthesia [7, 10, 14, 16], in the remaining 5 studies patients received either regional or general anesthesia [9, 11–13, 17], whereas in 2 studies no information regarding anesthetic method was provided (Supplementary material 3) [8, 15]. All patients underwent non-cardiac surgery [7–17]; however, in 2 studies, no “type of surgery” information was provided [7, 8]. 3 studies analyzed neurointerventional patients [9, 10, 16], 1 study analyzed liver transplantation patients [14], 1 study analyzed orthopedic surgery patients [11], whereas in the remaining 4 studies, patients underwent various non-cardiac procedures, of which abdominal and orthopedic surgeries were the most common [12, 13, 15, 17]. In regard to frequency of procedural electiveness, 5 studies included patients who underwent only elective surgery [7, 9, 10, 14, 16]; 5 studies had mixed populations of patients undergoing elective and non-elective procedures [8, 12, 13, 15, 17]. In one study, the data regarding emergent procedures were not provided (Supplementary material 3) [11].

**Table 1:** Summary of studies included for the analysis

Authors	Study type	Number of study participants	Intraoperative blood pressure variability	Outcomes
Neuner et al. [7]	Retrospective cohort	917	Blood pressure fluctuations were defined as the sum of the absolute differences between two consecutive measurements of systolic blood pressure during anesthesia	Higher intraoperative blood pressure variability was associated with POD
James et al. [8]	Retrospective cohort	1223	Number of episodes in which MAP changed ≥ 15% from the previous measurement	Higher intraoperative blood pressure variability was protective in regard to 30-day mortality
Zevallos et al. [9]	Case–control	33	Blood pressure variability defined as standard deviation and range	No statistically significant differences observed between CIN patients and controls
Li et al. [10]	Case–control	312	Blood pressure variability defined as average real variability (ARV)	Higher intraoperative blood pressure variability was associated with the early cerebral infarction
Radinovic et al. [11]	Prospective cohort	277	Blood pressure variability defined as a difference between the highest and the lowest mean arterial pressure that was measured intraoperatively (ΔMAP)	ΔMAP failed to be included in the multivariate model
Park et al. [12]	Retrospective cohort	Discovery cohort: 45,520 Validation cohort: 29,704	Blood pressure variability defined as Standard deviation (SD), Coefficient of variation (CV), Average real variability (ARV), Variation independent of the mean	Higher intraoperative blood pressure variability associated with postoperative AKI
Wiórek et al. [13]	Prospective cohort	835	Blood pressure variability defined as coefficient of variation (CV) of SBP, DBP, and MAP	Higher intraoperative blood pressure variability was associated with 30-day postoperative mortality
Prasad et al. [14]	Retrospective cohort	55	Blood pressure variability defined as median absolute deviation (MAD) of arterial blood pressure (ABP)	Higher blood pressure variability was protective against 180-day mortality
Mascha et al. [15]	Retrospective cohort	104,401	Blood pressure variability defined as MAP average real variability (ARV) or standard deviation	MAP-ARV presented a U-shaped relationship to the postoperative 30-day mortality. Lower intraoperative blood pressure variability was mildly associated with postoperative 30-day mortality
				MAP-SD presented a U-shaped relationship to the postoperative 30-day mortality. Lower and higher intraoperative blood pressure variability was mildly associated with postoperative 30-day mortality
Cai et al. [16]	Prospective cohort	2118	Maximum changes in BP were defined as the difference between highest and lowest mean arterial pressure (MAP) during surgery	Maximum change in blood pressure was independently associated with failed extubation
Levin et al. [17]	Retrospective cohort	Derivation cohort: 35,314 Validation cohort:17,605	Blood pressure variability defined as a number of percentage change between two consecutive MAP recordings	Higher blood pressure variability was independently associated with improved survival (in patients with no prior antihypertensive medications)

IBPV, intraoperative blood pressure variation; IQR, interquartile range; MAP, mean arterial pressure; SBP, systolic blood pressure; DBP, diastolic blood pressure; MAD, median absolute deviation; POD, postoperative delirium; OR, odds ratio, 95% CI, 95% confidence interval; SD, standard deviation; CV, coefficient of variation; ARV, average real variability

### Blood pressure variability definitions

Blood pressure component that was studied the most was the mean arterial pressure (*n* = 10) [8–17], then systolic blood pressure (*n* = 4) [7, 9, 10, 13] and diastolic blood pressure (*n* = 3) (Table 1) [9, 10, 13]. Blood pressure variability definitions varied between the studies: 5 studies implemented more than one definition of blood pressure variability [9, 10, 12, 13, 15], whereas the other 6 studies introduced only one definition [7, 8, 11, 14, 16, 17]. The most consistent definitions included a standard deviation (*n* = 4) [9, 12, 14, 15], a coefficient of variation (*n* = 2) [12, 13], an average real variability (*n* = 3) [10, 12, 15], and a variability defined as a difference between the highest and the lowest measured MAP (*n* = 2) [11, 16]. The remaining studies implemented different definitions [7, 8, 17] (Table 1).

### Outcomes

Mortality was the most frequently investigated outcome among the included studies (*n* = 6) [8, 12–15, 17], of which 30-day mortality was the most frequent (*n* = 4) [8, 13, 15, 17] (Table 1 and Supplementary material 3). The occurrence of 7-day postoperative delirium was described in 2 papers [7, 11]. The remaining studies focused on different aspects of postoperative complications, including acute kidney injury [12], contrast induced nephropathy [9], early cerebral infarction [10], and failed extubation [16].

### A relationship between IBPV and outcomes

In 5 studies, a relationship between higher intraoperative blood pressure variability and postoperative complications was observed [7, 10, 12, 13, 16]. In 3 studies, the authors discovered a protective effect of higher blood pressure variability on the risk of postoperative complications [8, 14, 17]. One study observed a U-shaped relationship between blood pressure variability and postoperative complications [15]. In the 2 remaining studies, no association between the variable and the outcome was observed [9, 10].

## Discussion

This systematic review focused on summarizing the data regarding intraoperative blood pressure variability and postoperative complications (as of April 2021). To our knowledge, this is the first attempt to systemize the body of evidence of the issue. No clear message regarding the nature of the relationship between IBPV and postoperative outcomes can be produced.

There were many different IBPV definitions applied across the studies. A standard deviation (SD) was the most frequently implemented definition of IBPV. SD reflects a spreadness of data from the mean value of all recordings; however, it does not take the mean value into account. An alternative then is to use a coefficient of variation (CV) which normalizes SD to the mean. For example, the same value of SD (e.g., 5) for two different means (10 and 100) results in dramatically different CVs: 50% and 5%. However, both CV and SD provide a global value of variation, irrespectively of measure-to-measure recordings. Hansel et al, provided an equation for an average real variability (ARV) which takes into account the latter [18]. They have shown that ARV can estimate blood pressure variability better than SD. Mascha et al., however, recognized that ARV is only valid in regard to equally distant time of measurements [15]. Therefore, they provided a generalized ARV formula which did not require equal time intervals between the recordings. ARV (and generalized ARV) was implemented in 3 studies. Due to above-mentioned reasons, we suggest using ARV as a standard measure of blood pressure variability. However, we are unable to make a clear recommendation regarding the physiological range of ARV as the authors present contrary results and, additionally, none of them provided a specific cut-off point for this parameter.

The studies of the best quality presented highly heterogeneous results [11, 12, 15, 17]. Therefore, no clear message regarding the relationship between IBPV between postoperative outcomes can be produced. The biggest methodological obstacle to overcome in regard to high variability is to understand the confounding effects of either hypo- or hypertension. Theoretically, the same IBPV values can result from two very different ranges of blood pressure, e.g., IBPV that occurs below hypotensive thresholds of blood pressure versus IBPV that occurs within physiological range of blood pressure. Indeed, in Park’s study of 29,704 patients (a validation cohort), a high IBPV was associated with postoperative AKI in a meaningful way only when the high variability occurred below 65 mmHg of MAP [12]. This finding contributes to the hypothesis that deeper variations (e.g., below certain thresholds) of BP may indeed be more harmful than little, more frequent IBPVs that would result in the same value of variability. Moreover, this could explain why in Wiórek’s, Cai’s, and Li’s studies, a higher degree of variability of MAP was associated with 30-day mortality: the authors did not adjust for intraoperative hypotension; therefore, a higher IBPV could reflect the occurrence of hypotension [10, 13, 16]. Nevertheless, a high IBPV, irrespective of the occurrence of hypotension, could result in perfusion disturbances, as rapid changes in blood pressure may exceed the capacity of adaptation and thus may not be simultaneously followed by sufficient neurohormonal and vascular response [12, 19]. Therefore, theoretically, frequent changes of blood pressure within the physiological range could still lead to imbalance of perfusion, albeit not exceeding hypotension thresholds. Two studies of the largest populations (Mascha = 104,401 patients, Levin = 52,919 patients), despite implementing different IBPV definitions, (ARV for Mascha and MAP lability > 10% for Levin) concluded that higher variability was associated with 30-day postoperative survival [13, 15]. Interestingly, in Mascha’s study, they observed a U-shaped relationship between ARV and mortality. By taking into account a number of potentially confounding factors (including time-weighted average MAP), they provided conclusions that low IBPV was associated with 30-day mortality and that low IBPV could reflect the impaired circulatory system’s ability to adapt to the hemodynamic disturbances, and thus, patients with low IBPV would be more susceptible to such alterations and be unable to restore homeostasis, and, therefore, have a higher risk of insufficient perfusion. Such a hypothesis was based on considerations that low heart rate variability is a marker of autonomic dysfunction in patients with heart failure and could predict cardiovascular events [15, 20]. Moreover, anesthesia depth may play a role in shaping the IBPV as excessive doses of anesthetic agents interfere with the autonomic system response, often leading to sympathetic depression [21]. It is likely that depth of anesthesia influences hemodynamic stability; however, as of now, there are no studies assessing this issue in regard to postoperative complications.

In terms of limitations, the data covered by this review are very heterogeneous in regard to study populations, IBPV definitions, and outcomes. Therefore, we failed to prepare a reliable meta-analysis. Secondly, the studies varied in terms of quality of reporting. Thirdly, all the studies included in the review were observational studies; therefore, we cannot imply causality of IBPV on postoperative outcomes. Taking into account all of the above-mentioned considerations, we believe that unification of IBPV definition is the first step to ultimately determine the role of IBPV. Further studies exploring this parameter, especially within the physiological BP thresholds, could expand our knowledge regarding its influence on perfusion disturbances.

## Conclusion

To conclude, based on a limited number of studies, IBPV does not seem to be a reliable indicator in predicting postoperative complications. The primary question of this systematic review cannot be clearly answered. Existing premises suggest that, under different circumstances, either higher or lower IBPV could contribute to postoperative complications. Taking into account the heterogeneity and quality of the studies, the conclusions may not be definitive and further well-designed studies are needed to clarify this unclear relationship.

## Supplementary Information

Below is the link to the electronic supplementary material.

Supplementary file1 (DOCX 16 KB)

Supplementary file2 (DOCX 17 KB)

Supplementary file3 (DOCX 20 KB)
