# Supplementary material for: The impact of intraoperative blood pressure variability on the risk of postoperative adverse outcomes in non-cardiac surgery: a systematic review
Source: J Anesth. 2022 Jan 13;36(2):316–22. doi: 10.1007/s00540-022-03035-w (PMC8967760; doi:10.1007/s00540-022-03035-w)
Supplement: Supplementary file 1 — Supplementary file1 (DOCX 16 KB) [file 540_2022_3035_MOESM1_ESM.docx]

**Search strings**

**PubMed:** (((intraoperative OR intraoperatively OR perioperative OR perioperatively) AND (variation OR lability OR variability OR deviation OR coefficient of variation OR fluctuation)) AND (myocardial injury OR major adverse cerebrovascular cardiovascular events OR postoperative complication* OR adverse outcome* OR cardiac OR renal OR organ injury OR organ dysfunction OR acute kidney injury OR myocardial infarction OR stroke OR death OR mortality OR length of stay OR cerebral OR complication OR adverse event* OR ischaem* OR injury OR delirium OR cerebrovascular OR coronary OR LOS OR accident)) AND (blood pressure OR systolic blood pressure OR diastolic blood pressure OR mean arterial pressure)

**MeSH:** (((((((((((("Perioperative Period"[Mesh]) AND "Blood Pressure"[Mesh]) AND "Acute Kidney Injury"[Mesh])) OR "Mortality"[Mesh]) OR "Delirium"[Mesh]) OR "Length of Stay"[Mesh]) OR "Ischemia"[Mesh]) OR "Stroke"[Mesh]) OR "Multiple Organ Failure"[Mesh]) OR "Myocardial Ischemia"[Mesh])) AND "Biological Variation, Individual"[Mesh]

**EMBASE:**(intraoperative OR intraoperatively OR perioperative OR perioperatively) AND ((variation OR lability OR variability OR coefficient) AND of AND variation OR fluctuation OR deviation) AND (((((((((myocardial AND injury OR major) AND adverse AND cerebrovascular AND cardiovascular AND events OR postoperative) AND complication* OR adverse) AND outcome OR cardiac OR renal OR organ) AND dysfunction OR acute) AND kidney AND injury OR myocardial) AND infarction OR stroke OR death OR mortality OR length) AND of AND stay OR cerebral OR complication OR adverse) AND event* OR ischaem* OR injury OR delirium OR cerebrovascular OR coronary OR los OR accident) AND (((blood AND pressure OR systolic) AND blood AND pressure OR diastolic) AND blood AND pressure OR mean) AND arterial AND pressure

**Web of Science: intraoperative OR intraoperatively OR perioperative OR perioperatively (All Fields) and variation OR lability OR variability OR deviation OR coefficient of variation OR fluctuation (All Fields) and myocardial injury OR major adverse cerebrovascular cardiovascular events OR postoperative complication* OR adverse outcome* OR cardiac OR renal OR organ injury OR organ dysfunction OR acute kidney injury OR myocardial infarction OR stroke OR death OR mortality OR length of stay OR cerebral OR complication OR adverse event* OR ischaem* OR injury OR delirium OR cerebrovascular OR coronary OR LOS OR accident (All Fields) and blood pressure OR systolic blood pressure OR diastolic blood pressure OR mean arterial pressure (All Fields)**

**Clinicaltrials.gov:** blood pressure variability OR blood pressure variation OR blood pressure coefficient of variation OR blood pressure fluctuation OR blood pressure deviation | Studies With Results | Observational Studies

## SCOPUS:( ALL ( intraoperative  OR  intraoperatively  OR  perioperative  OR  perioperatively )  AND  ALL ( variation  OR  lability  OR  variability  OR  deviation  OR  coefficient  AND of  AND variation  OR  fluctuation )  AND  ALL ( myocardial  AND injury  OR  major  AND adverse  AND cerebrovascular  AND cardiovascular  AND events  OR  postoperative  AND complication*  OR  adverse  AND outcome*  OR  cardiac  OR  renal  OR  organ  AND injury  OR  organ  AND dysfunction  OR  acute  AND kidney  AND injury  OR  myocardial  AND infarction  OR  stroke  OR  death  OR  mortality  OR  length  AND of  AND stay  OR  cerebral  OR  complication  OR  adverse  AND event*  OR  ischaem*  OR  injury  OR  delirium  OR  cerebrovascular  OR  coronary  OR  los  OR  accident )  AND  ALL ( blood  AND pressure  OR  systolic  AND blood  AND pressure  OR  diastolic  AND blood  AND pressure  OR  mean  AND arterial  AND pressure ) )

## Cochrane Library: intraoperative OR intraoperatively OR perioperative OR perioperatively in Title Abstract Keyword AND variation OR lability OR variability OR deviation OR coefficient of variation OR fluctuation in Title Abstract Keyword AND myocardial injury OR major adverse cerebrovascular cardiovascular events OR postoperative complication* OR adverse outcome* OR cardiac OR renal OR organ injury OR organ dysfunction OR acute kidney injury OR myocardial infarction OR stroke OR death OR mortality OR length of stay OR cerebral OR complication OR adverse event* OR ischaem* OR injury OR delirium OR cerebrovascular OR coronary OR LOS OR accident in Title Abstract Keyword AND blood pressure OR systolic blood pressure OR diastolic blood pressure OR mean arterial pressure in Title Abstract Keyword - in Cochrane Reviews, Trials, Special Collections (Word variations have been searched)
