# Supplementary material for: The impact of intraoperative blood pressure variability on the risk of postoperative adverse outcomes in non-cardiac surgery: a systematic review
Source: J Anesth. 2022 Jan 13;36(2):316–22. doi: 10.1007/s00540-022-03035-w (PMC8967760; doi:10.1007/s00540-022-03035-w)
Supplement: Supplementary file 2 — Supplementary file2 (DOCX 17 KB) [file 540_2022_3035_MOESM2_ESM.docx]

Table 1. Newcastle-Ottawa scale quality assessment

| **Author** | **Study type** | **S1** | **S2** | **S3** | **S4** | **C1A** | **C1B** | **O1** | **O2** | **O3** | **Total** |
| --- | --- | --- | --- | --- | --- | --- | --- | --- | --- | --- | --- |
| Neuner et al. (2016) [7] | Retrospective cohort | 1 | 1 | 1 | 1 | 0 | 0 | 1 | 1 | 1 | 7/9  POOR |
| James et al. (2019) [8] | Retrospective cohort | 1 | 1 | 1 | 1 | 0 | 0 | 1 | 1 | 0 | 6/9 POOR |
| Zevallos et al. (2021) [9] | Case-control | 1 | 0 | 1 | 1 | 0 | 0 | 1 | 1 | 1 | 6/9 POOR |
| Li et al. (2020) [10] | Case-control | 1 | 0 | 1 | 1 | 0 | 0 | 1 | 1 | 1 | 6/9 POOR |
| Radinovic et al. (2020) [11] | Prospective cohort | 1 | 1 | 1 | 1 | 1 | 1 | 1 | 1 | 1 | 9/9 GOOD |
| Park et al. (2020) [12] | Retrospective cohort | 1 | 1 | 1 | 1 | 1 | 1 | 1 | 1 | 0 | 8/9 GOOD |
| Wiórek et al. (2019) [13] | Prospective cohort | 1 | 1 | 1 | 1 | 0 | 0 | 1 | 1 | 1 | 7/9 POOR |
| Prasad et al. (2015) [14] | Retrospective cohort | 0 | 1 | 1 | 1 | 0 | 0 | 1 | 1 | 0 | 5/9 POOR |
| Mascha et al. (2015) [15] | Retrospective cohort | 1 | 1 | 1 | 1 | 1 | 1 | 1 | 1 | 1 | 9/9 GOOD |
| Cai et al. (2016) [16] | Prospective cohort | 0 | 1 | 1 | 1 | 0 | 0 | 1 | 1 | 1 | 6/9 POOR |
| Levin et al. (2015) [17] | Retrospective cohort | 1 | 1 | 1 | 1 | 1 | 1 | 1 | 1 | 1 | 9/9 GOOD |

Legend:S1: Selection: Representativeness of the exposed cohort; S2: Selection of the non-exposed cohort; S3: Ascertainment of exposure; S4: Demonstration that outcome of interest was not present at start of study; C1A: Comparability of cohorts on the basis of the design or analysis controlled for confounders: the study controls for gender of a relative; C1B: Comparability of cohorts on the basis of the design or analysis controlled for confounders: the study controls for other factors; O1: Assessment of outcome; O2: Was follow-up long enough for outcomes to occur; O3: Adequacy of follow-up of cohorts. The quality of a study was assessed by converting Newcastle-Ottawa scale to AHRQ standard (good, fair and poor): Good quality: 3 or 4 stars in selection domain AND 1 or 2 stars in comparability domain AND 2 or 3 stars in outcome/exposure domain; Fair quality: 2 stars in selection domain AND 1 or 2 stars in comparability domain AND 2 or 3 stars in outcome/exposure domain; Poor quality: 0 or 1 star in selection domain OR 0 stars in comparability domain OR 0 or 1 stars in outcome/exposure domain
