# Supplementary material for: The impact of intraoperative blood pressure variability on the risk of postoperative adverse outcomes in non-cardiac surgery: a systematic review
Source: J Anesth. 2022 Jan 13;36(2):316–22. doi: 10.1007/s00540-022-03035-w (PMC8967760; doi:10.1007/s00540-022-03035-w)
Supplement: Supplementary file 3 — Supplementary file3 (DOCX 20 KB) [file 540_2022_3035_MOESM3_ESM.docx]

Table 1. Summary of studies included for the analysis.

| **Authors** | **Study type** | **Study population** | **Intraoperative blood pressure variability** | **End-point** | **Outcomes** |
| --- | --- | --- | --- | --- | --- |
| Neuner et al. (2016) [7] | Retrospective cohort | 917 patients (male=53.3%) of mean age 70 ± 6.5 years who underwent non-cardiac surgery. General anaesthesia was present in 100% cases. | Blood pressure fluctuations were defined as the sum of the absolute differences between two consecutive measurements of systolic blood pressure during anaesthesia. Blood pressure measurement intervals: 1 minute | Cumulative postoperative delirium (POD) incidence within 7 postoperative days | **Multivariate analysis:**  Higher intraoperative blood pressure variability was associated with POD  OR=1.025  (CI 95% 1.004-1.045): p<0.0001 for fluctuations in systolic blood pressure, per 10 mmHg |
| James et al. (2019) [8] | Retrospective cohort | 1223 patients (male=46.7%) of mean age 75.32 ± 7.23 years who underwent non-cardiac surgery. | Number of episodes in which MAP changed ≥ 15% from the previous measurement.  Blood pressure measurement intervals: 5 minutes | 30-day mortality | **Multivariate analysis:**  Higher intraoperative blood pressure variability was protective in regards to 30-day mortality  OR=0.928 (CI 95% 0.862-1.000); p=0.049 for fractional change of MAP ≥ 15%, per 1 episode |
| Zevallos et al. (2021) [9] | Case-control | 11 patients with contrast induced neuropathy (CIN) of median age of 76 years and 22 patients matched for control of median age of 66 years (male=33.3%) undergoing neurointerventional procedures (82% unruptured cerebral aneurysm and 18% carotid stenosis). 78% of patients received general anaesthesia. | Blood pressure variability defined as standard deviation and range.  Blood pressure measurement intervals: 2-5 minutes | Difference in blood pressure variability between patients with CIN and controls | No statistically significant differences o observed between CIN patients and controls:  SBP range: 59 vs 56 (median); p=0.97  SBP SD: 13 vs 11 (median); p=0.375  DBP range: 22 vs 36 (median); p=0.311  DBP SD: 4 vs 6 (median); p=0.44  MAP range: 22 vs 36 (median); p=0.462  MAP SD: 8 vs 7 (median); p=0.807 |
| Li et al. (2020) [10] | Case-control | Patients with Moyamoya disease: 52 patients with early cerebral infarction of mean age of 38.46 ± 11.70 years and 260 controls of mean age of 39.18 ± 11.57 years (male=47.7%) undergoing revascularization surgery. General anaesthesia was present in 100% cases. | Blood pressure variability defined as average real variability (ARV).  Blood pressure measurement intervals: 5 minutes | Difference in blood pressure variability between patients with early cerebral infarction and controls | **Multivariate analysis  (cases vs control):**  Higher intraoperative blood pressure variability was associated with the early cerebral infarction  -ARV-SBP (per 1 mmHg/min): 1.25 vs 1.05; OR=3.18 (CI99% 1.32-10.30); p=0.002  -ARV-DBP (per 1 mmHg/min):0.93 vs 0.82; OR=4.04 (CI99% 1.04-16.82); p=0.006  -ARV-MAP (per 1 mmHg/min):0.93 vs 0.80; OR=4.02 (CI99% 1.22-17.46); p=0.004 |
| Radinovic et al. (2020) [11] | Prospective cohort | 277 patients (of mean age 78 years) undergoing hip fracture surgery with either general anaesthesia (56%) or regional anaesthesia (44%) | Blood pressure variability defined as a difference between the highest and the lowest mean arterial pressure that was measured intraoperatively (ΔMAP).  Blood pressure measurement intervals: 5 minutes | The occurrence of POD during the first 7 days after surgery | **Univariate analysis:**  ΔMAP was higher in patients who developed POD than in those who did not develop POD (16.7 vs 13.3; p=0.007)  **Multivariate analysis**:  ΔMAP failed to be included in the multivariate model |
| Park et al. (2020) [12] | Retrospective cohort | **1** **(discovery cohort)** - 45520 patients (median age 56 years) who underwent general anaesthesia in 86% cases  **2 (validation cohort)** - 29704 patients (male=53.0%) of median age 61 years who underwent general anaesthesia in 76% cases.    Patients underwent various non-cardiac surgeries of which general surgery was the most common (~40%). | Blood pressure variability defined as:  Standard deviation (SD),  Coefficient of variation (CV),  Average real variability (ARV),  Variation independent of the mean.  Blood pressure measurement intervals: either continuous or 1-5 minutes. | The occurrence of postoperative acute kidney injury (AKI) and critical AKI (stage 2 or higher AKI and post-AKI death or dialysis within 90 days) | **Multivariate analysis  (validation cohort):**  Higher intraoperative blood pressure variability associated with postoperative AKI  **1 - postoperative AKI:**  -MAP-SD (per 3.7 mmHg): OR=1.12  (CI 95%1.05-1.20); p<0.001  -MAP-CV (per 4.4%): OR=1.14  (CI95% 1.06-1.22); p<0.001  -MAP-ARV (per 3.1 mmHg/measurement):  1.10 (95%CI 1.03-1.17); p<0.001  - MAP-Variation independent of mean (per 0.08): OR=1.13 (CI95% 1.06-1.21); p<0.001  **2 - postoperative critical AKI**  -MAP-SD (per 3.7 mmHg): OR=1.17  (CI 95%1.03-1.31); p=0.01  -MAP-CV (per 4.4%): OR=1.16 (CI95% 1.02-1.31); p=0.02  -MAP-ARV (per 3.1 mmHg/measurement):  1.25 (95%CI 1.12-1.39); p<0.001  -MAP-Variation independent of mean (per 0.08): OR=1.16 (CI95% 1.03-1.31); p=0.02 |
| Wiórek et al. (2019) [13] | Prospective cohort | 835 patients (male=27.7%) of median age 48 years undergoing various non-cardiac procedures (26% gastrointestinal, 44% gynecological, 30% neurosurgical) of which 16.4% were emergency ones. General anaesthesia was present in 77% of cases. | Blood pressure variability defined as coefficient of variation (CV) of SBP, DBP and MAP.  Blood pressure measurement intervals: 5 minutes | Postoperative 30-day mortality | **Multivariate analysis:**  Higher intraoperative blood pressure variability was associated with 30-day postoperative mortality  -CV-SBP (per 1%): OR=1.10 (95%CI 1.00-1.21); p=0.05  -CV-DBP (per 1%): OR=1.10 (95%CI 1.01-1.21);p=0.03  -CV- MAP (per 1%): OR=1.10 (95%CI 0.99-1.23); p=0.06 |
| Prasad et al. (2015) [14] | Retrospective cohort | 55 patients of mean age of 55 ± 6 years  undergoing orthotopic liver transplantation | Blood pressure variability defined as median absolute deviation (MAD) of arterial blood pressure (ABP).  Blood pressure measurement intervals: continuous | 180-day mortality after surgery | **Multivariate logistic regression**:  Higher blood pressure variability was protective against 180-day mortality  MAD of systolic ABP (per 1 mmHg): OR= 0.696 (95%CI 0.506-0.957); p=0.026 |
| Mascha et al. (2015) [15] | Retrospective cohort | 104,401 patients (male=53%) of mean age ~ 57 years undergoing noncardiac surgery  (~5% of the procedures were emergency surgeries) | Blood pressure variability defined as MAP average real variability (ARV) or standard deviation.  Blood pressure measurement intervals: 1-5 minutes | Postoperative 30-day mortality | **Multivariate analysis:**  MAP-ARV presented a U-shaped relationship to the postoperative 30-day mortality. Lower intraoperative blood pressure variability was mildly associated with postoperative 30-day mortality  - median ARV= 2.3: OR= 1.0 (reference)  -for 25th percentile of ARV (ARV= 1.6 mmHg/min): OR=1.14 (95%CI 1.03-1.25); p =0.01  -for 75th percentile of ARV (ARV= 3.6 mmHg/min): OR=0.94 (CI95% 0.88-0.99); p=0.018  MAP-SD presented a U-shaped relationship to the postoperative 30-day mortality. Lower and higher intraoperative blood pressure variability was mildly associated with postoperative 30-day mortality  -for 25th percentile of SD: OR=1.09 (95%CI 1.03-1.16); p=0.006  -for 75th percentile of SD: OR=1.05 (CI95% 1.01-1.10); p=0.033 |
| Cai et al. (2016) [16] | Prospective cohort | 2118 patients (male=38.7%) of mean age 45.5±13.4 years who received infratentorial craniotomy for brain tumor resection. General anaesthesia was present in 100% cases. | Maximum changes in BP were defined as the difference between highest and lowest mean arterial pressure (MAP) during surgery.  Blood pressure measurement intervals: unclear | Failed extubation defined as the need for reintubation or tracheotomy within 72 hours following the scheduled extubation for any reasons other than reintubation or tracheostomy for reoperation | **Multivariate analysis:**  Maximum change in blood pressure was independently associated with failed extubation    Maximum change in MAP (per 1 mmHg):OR=1.039 (95% CI 1.024- 1.055); p<0.001 |
| Levin et al. (2015) [17] | Retrospective cohort | 52919 adult patients undergoing various non-cardiac surgeries (general surgery as the most common type) undergoing general anaesthesia (83.6% patients in both cohorts). 9% of all procedures were emergency ones:  **1 (derivation cohort)** -  35314 patients (median age 58 years)  **2 (validation cohort) -**  17605 patients (male=46.5%) of median age 57 years | Blood pressure variability defined as a number of percentage change between two consecutive MAP recordings.  Blood pressure measurement intervals: either continuous or 1-5 minutes. | Postoperative 30-day mortality | **Multivariate analysis**  **(validation cohort):**  Higher blood pressure variability was independently associated with improved survival (in patients with no prior antihypertensive medications)  MAP lability> 10% (per 1 episode):  OR=0.96 (95%CI 0.93-0.99); p=0.01 |

IBPV: intraoperative blood pressure variation; IQR: interquartile range; MAP: mean arterial pressure; SBP: systolic blood pressure; DBP: diastolic blood pressure;MAD: median absolute deviation; POD: postoperative delirium; OR: odds ratio, 95% CI: 95% confidence interval; SD: standard deviation; CV: coefficient of variation; ARV: average real variability
